# A note on obtaining correct marginal predictions from a random intercepts model for binary outcomes

**DOI:** 10.1186/s12874-015-0046-6

**Published:** 2015-08-05

**Authors:** Menelaos Pavlou, Gareth Ambler, Shaun Seaman, Rumana Z. Omar

**Affiliations:** Department of Statistical Science, University College London, Gower St., London, WC1E 6BT UK; MRC Biostatistics Unit, Cambridge, UK

**Keywords:** Random effects model, Marginal predictions, Calibration

## Abstract

**Background:**

Clustered data with binary outcomes are often analysed using random intercepts models or generalised estimating equations (GEE) resulting in cluster-specific or ‘population-average’ inference, respectively.

**Methods:**

When a random effects model is fitted to clustered data, predictions may be produced for a member of an existing cluster by using estimates of the fixed effects (regression coefficients) and the random effect for the cluster (conditional risk calculation), or for a member of a new cluster (marginal risk calculation). We focus on the second. Marginal risk calculation from a random effects model is obtained by integrating over the distribution of random effects. However, in practice marginal risks are often obtained, incorrectly, using only estimates of the fixed effects (i.e. by effectively setting the random effects to zero). We compare these two approaches to marginal risk calculation in terms of model calibration.

**Results:**

In simulation studies, it has been seen that use of the incorrect marginal risk calculation from random effects models results in poorly calibrated overall marginal predictions (calibration slope <1 and calibration in the large ≠ 0) with mis-calibration becoming worse with higher degrees of clustering. We clarify that this was due to the incorrect calculation of marginal predictions from a random intercepts model and explain intuitively why this approach is incorrect. We show via simulation that the correct calculation of marginal risks from a random intercepts model results in predictions with excellent calibration.

**Conclusion:**

The logistic random intercepts model can be used to obtain valid marginal predictions by integrating over the distribution of random effects.

**Electronic supplementary material:**

The online version of this article (doi:10.1186/s12874-015-0046-6) contains supplementary material, which is available to authorized users.

## Background

Clustered data arise often in medical research, for example when patients are clustered within hospitals or general practises. It is well known [1] that a standard regression model which ignores clustering provides incorrect standard errors for the estimates of the regression coefficients. Random effects models (also known as ‘mixed models’) and Generalised Estimating Equations (GEE) are two popular approaches for analysing clustered data; they account for clustering and provide, respectively, cluster-specific (conditional) and population-average (marginal) inference [2]. For a continuous outcome, the fit from a linear regression mixed model and GEE estimate the same population quantities (regression coefficients), but for a binary outcome a logistic regression mixed model and GEE estimate different quantities [1]. In this paper we focus on binary outcomes.

For example, Bouwmeester et al. [3] focused on the use of a logistic mixed model with random intercepts to obtain conditional risk predictions using estimates of the fixed and random effects. This type of prediction may be used to predict the probability of an event for a new member of an existing cluster, i.e. a cluster that belongs to the dataset that was used to fit the random effects model. They found that conditional risk predictions provided better discrimination than marginal risk predictions. They used the same random intercepts model to produce marginal predictions, by simply setting the random effects to zero. Skrondal et al. [4] had earlier noted that this incorrect type of marginal prediction is not unusual in applied research (e.g. Rose [5] ), and was shown to result in a small loss in predictive accuracy when the intra-cluster correlation (ICC) was small. Bouwmeester et al. [3] used the incorrect marginal risk calculation and found that it produced mis-calibrated predictions with ICC as small as 0.15. The amount of mis-calibration was found to increase with the degree of clustering.

We clarify the reasons for this mis-calibration and explain how to obtain correct marginal predictions from a logistic random intercepts model.

## Methods

### Marginal predictions for clustered data

Let *Y*_*ij*_ and ***X***_*ij*_ = (*X*_*ij*,1_, *X*_*ij*,2_, …, *X*_*ij*,*p*_) denote the binary outcome and the *p*-dimensional vector of covariates for the *j*^*th*^ member of the *i*^*th*^ cluster, *i* = 1, …, *K*; *j* = 1, …, *N*_*i*_.

A logistic random intercepts model can be written as:

1 $$ \mathrm{logit}\ \left(P\left({Y}_{ij}=1\Big|{\boldsymbol{X}}_{ij}={\boldsymbol{x}}_{ij},{u}_i\right)\right)={\alpha}_{RE}+{u}_i+{\displaystyle \sum_{m=1}^p}{x}_{ij,m}\ {\beta}_{RE,m}, $$

where *a*_*RE*_ and ***β***_*RΕ*_ = (*β*_*RΕ*,1_, *β*_*RΕ*,2_, …, *β*_*RΕ*,*m*_) are the *conditional* regression parameters (fixed effects), and *u*_*i*_ is the random effect for the *i*^*th*^ cluster. Usually it is assumed that *u*_*i*_ ~ *N*(0, *σ*_*u*_^2^).

The linear combination of predictor values, regression coefficients and random effect, ${\alpha}_{RE}+{u}_i+{\displaystyle \sum_{m=1}^p}{x}_{ij,m}{\beta}_{RE,m}$, is known as linear predictor, risk score or predicted log-odds.

A marginal model (not conditional on random effects) can be written as:

2 $$ \mathrm{logit}\left(P\left({Y}_{ij}=1\Big|{\boldsymbol{X}}_{ij}={\boldsymbol{x}}_{ij}\right)\right)={\alpha}_M+{\displaystyle \sum_{m=1}^p}{x}_{ij,m}\ {\beta}_{M,m}, $$

where *a*_*M*_ and ***β***_*M*_ = (*β*_*M*,1_, *β*_*M*,2_, …, *β*_*M*,*m*_) are the *marginal* regression coefficients. Model (2) can be fitted using standard Maximum Likelihood Estimation (MLE) ignoring the clustering. This results in estimators ${\widehat{\alpha}}_{\mathrm{standard}}$ and ${\widehat{\boldsymbol{\beta}}}_{\mathrm{standard}}$.

Ideally, the within-cluster correlation should be accounted for, and model (2) be fitted using GEE with a suitable ‘working’ correlation matrix, giving rise to estimators ${\widehat{\alpha}}_{\mathrm{GEE}}$ and ${\widehat{\boldsymbol{\beta}}}_{\mathrm{GEE}}$. Both $\left({\widehat{\alpha}}_{\mathrm{standard}},{\widehat{\boldsymbol{\beta}}}_{\mathrm{standard}}\right)$ and $\left({\widehat{\alpha}}_{\mathrm{GEE}},{\widehat{\boldsymbol{\beta}}}_{\mathrm{GEE}}\right)$ are consistent estimates of (*α*_*M*_, ***β***_*M*_) (i.e. converge to the true value (*α*_*M*_, ***β***_*M*_) as the sample size tends to infinity) provided that any missing data are covariate-dependent MCAR (Missing Completely at Random) [6]. Note that $\left({\widehat{\alpha}}_{\mathrm{standard}},{\widehat{\boldsymbol{\beta}}}_{\mathrm{standard}}\right)=\left({\widehat{\alpha}}_{\mathrm{GEE}},{\widehat{\boldsymbol{\beta}}}_{\mathrm{GEE}}\right)$ when the independence working correlation matrix is used.

Importantly, Zeger et al. [7] noted that the fixed effects in a random intercepts model are larger (in absolute value) than the corresponding regression parameters from a marginal model. That is,

3 $$ \left|{a}_{RE}\left|\ge \Big|{a}_M\right|,\ \right|{\beta}_{RE,m}\left|\ge \Big|{\beta}_{M,m}\right|\ \forall\ m, $$

with the equality holding if and only if *a*_*RE*_ = 0 or *β*_*RE,m*_ = 0.

Also, the difference between the marginal and conditional coefficients increases as the variance of the random effects (i.e. the degree of clustering) increases. In particular, an approximation [7] of (*α*_*M*_, ***β***_*M*_) from (*α*_*RE*_, ***β***_*RE*_) is

4 $$ {\alpha}_{M,m} \approx \frac{\alpha_{RE,m\ }}{\sqrt{\ \left({c}^2{\sigma}_u^2+1\right)}},\ {\beta}_{M,m} \approx \frac{\beta_{RE,m\ }}{\sqrt{\ \left({c}^2{\sigma}_u^2+1\right)}},\ c=\frac{16\sqrt{3}}{15\pi }\ . $$

Bouwmeester et al. [3] calculated marginal risk predictions from the random intercepts model using *only* the fixed effects, i.e. by setting the random effects equal to their mean value, zero:

5 $$ P\left({Y}_{ij}=1\Big|{\boldsymbol{X}}_{ij}={\boldsymbol{x}}_{ij}\right)={\widehat{\pi}}_{ij}(0)=\frac{1}{1+ \exp \left(-\left[{\widehat{a}}_{RE}+{\displaystyle {\sum}_{m=1}^p}{x}_{ij,m}\ {\widehat{\beta}}_{RE,m}\right]\ \right)}\ . $$

However, as noted by Skrondal et al. [4] the correct way to obtain marginal risk predictions from a random intercepts model is to integrate over the distribution of random effects:

6 $$ P\left({Y}_{ij}=1\Big|{\boldsymbol{X}}_{ij}={\boldsymbol{x}}_{ij}\right)={\widehat{\pi}}_{ij}(int)={\displaystyle \underset{-\infty }{\overset{\infty }{\int }}}\frac{1}{1+ \exp \left(-\left[{\widehat{a}}_{RE}+u+{\displaystyle {\sum}_{m=1}^p}{x}_{ij,m}\ {\widehat{\beta}}_{RE,m}\ \right]\right)}f(u)\ du, $$

where *f*(*u*) is the density function of the (prior) distribution of random effects. In this case it is the density function for a Normal distribution with mean zero and variance *σ*_*u*_^2^, which can be substituted by its estimate, ${\widehat{\sigma}}_u^2$.

We next design a simulation study to compare in terms of calibration the following 5 types of marginal predictions from:

- a marginal regression model (fitted using standard maximum likelihood) that ignores clustering (‘No clustering’).
- a random intercepts regression model with the random effects set to zero (‘RE-zero’).
- a random intercepts regression model after integrating over the estimated distribution of random effects (‘RE-integ’),
- a random intercepts regression model after rescaling the estimated conditional coefficients using the approximation of Zeger (‘RE-approx’),
- a marginal regression model fitted by GEE with exchangeable correlation structure (‘GEE’).

### Simulation study

We designed a simulation to demonstrate that the correct approach to obtaining marginal predictions from a random intercepts model using equation (6) results in predictions with good calibration, regardless of the degree of clustering. We replicated the simulation study of Bouwmeester et. al. [3] (however, since the random number seed used in their simulation study was unknown to us, we have obtained slightly different results from those shown in their tables).

The simulation design was as follows. A source population of 100 centres was generated with varying cluster size, *N*_*i*_, for each cluster. In particular, *N*_*i*_ ~ *Poisson*(exp(*λ*_*i*_)), *λ*_*i*_ ~ *N*(5.7, 0.3^2^), *i* = 1, …, 100. The total sample comprised 32502 patients and the median number of patients per centre was 312.

Six predictor variables were considered, three of which were continuous and were generated from Normal distributions with zero mean and standard deviations 1, 0.3, 0.2, respectively. The other three predictors were binary with prevalence 0.2, 0.3, 0.4, respectively, and were generated from a binomial distribution. The coefficients of all predictors were set to 1. The degree of clustering was varied by changing the variance *σ*_*u*_^2^ of a latent variable *u* ~ *N*(0, *σ*_*u*_^2^). The ICC is given by $ICC=\frac{\sigma_u^2}{\pi^2/3+{\sigma}_u^2}$ [8], and so when *σ*_*u*_^2^ = 0.17, ICC = 0.05. The linear predictor and the probability of an event happening, respectively, were calculated using

$$ {\eta}_{ij}={u}_i+{x}_{ij,1}+{x}_{ij,2}+{x}_{ij,3}+{x}_{ij,4}+{x}_{ij,5}+{x}_{ij,6}\mathrm{and}\kern0.5em P\left({Y}_{ij}=1\right)={\left(1+ \exp \left(-{\eta}_{ij}\right)\right)}^{-1} $$

Finally, the binary outcome *Y*_*ij*_ was randomly set equal to 1 with probability *P*(*Y*_*ij*_ = 1) and 0 otherwise.

Study samples were drawn from this source population using a two-stage sampling. First, 20 centres were drawn from the population of 100 clusters, and then 1000 members were drawn at random from the sampled centres. This two stage sampling procedure was repeated 100 times so 100 ‘training’ datasets were produced. The source population, each time after excluding the 20 clusters that were sampled for the training dataset, was taken to be the ‘test’ or ‘validation’ dataset for the corresponding training dataset. For each method for calculating marginal risks, the model was fitted to each of the training datasets, and its predictive performance was assessed on the corresponding validation dataset.

In addition, to investigate the sensitivity of the performance of the methods to differences between the distributions of cluster sizes in the training and validation datasets, we also considered a second method for constructing the training datasets. In this second method, each training dataset was generated using the same distribution of predictors as in the validation dataset, but the distribution of cluster sizes differed. In particular, each training dataset consisted of a total of 1000 observations in 20 clusters, and the cluster sizes were generated from a multinomial distribution with probabilities generated from a Dirichlet distribution with parameter equal to one for all clusters.

We examined the predictive performance of different methods to obtain marginal risks, in terms of calibration. Calibration in the large and calibration slope are calculated by fitting to the validation data two separate regression models. Calibration slope is the slope term in a logistic regression model where the estimated linear predictor (the inverse logit function of the predicted probability, also known as risk score and predicted log-odds) is regressed on the binary outcome. Calibration in the large is the intercept term in an intercept-only logistic regression model for the binary outcome with the linear predictor set as an offset term. A perfectly calibrated model should have a calibration in the large value equal to 0 (meaning that the average predicted probability is equal to the prevalence of the outcome) and a calibration slope value equal to 1. A calibration slope <1 indicates that the range of predictions is excessively wide and some shrinkage is necessary [9].

The simulations were carried out using R version 3.1. The logistic random intercepts models were fitted using the glmer function of the R package lme4. Population-average predictions, via numerical integration over the estimated distribution of random effects, were obtained by user-written code (see Additional file 1). The same computation can be carried out in Stata using built-in commands of the package gllamm.

## Results

We now discuss the results of using the wrong form described by equation (5) for marginal predictions. Table 1 presents calibration in the large and calibration slopes (with standard errors) for the 5 methods above and for varying degrees of clustering. Use of marginal predictions by setting the random effects equal to zero (RE-zero) results in increasingly poorly calibrated predictions as the ICC becomes higher. For the highest ICC considered, ICC = 0.3, the calibration in the large is 0.14, which means that the average predicted risk was too low, and the calibration slope is 0.79, which indicates that the predicted risks are more widely dispersed than the actual risks. A calibration slope of less than one can be a symptom of overfitting [10] and in practice, values slightly less than one are expected due to minor overfitting. However, overfitting as a cause is ruled out here because in this simulation the number of events was large. Instead, it can be purely attributed to the incorrect approach used to obtain marginal predictions from a random intercepts model. As the regression coefficients of a random intercepts model are larger than the corresponding marginal ones, by just setting the random effects to zero we obtain an excessively wide range of predictions which leads to poor calibration slope (<1). Similarly, the mean of the predicted risks ${\widehat{\pi}}_{ij}(0)$ is not equal to the mean of the predicted risks ${\widehat{\pi}}_{ij}(int)$, which leads to poor calibration in the large (≠ 0). Note that the larger the intra-cluster correlation is, the larger is the ratio of |*β*_*RE*,*m*_| to |*β*_*M*,*m*_|, as can be easily seen from the approximation in (4). So, we would expect that for an ICC smaller than 0.3, the calibration slope for RE-zero would be closer to 1. Indeed, the results in Table 1, verify this intuition: when ICC = 0.05, RE-zero has a calibration slope of 0.95 and calibration in the large -0.03.

**Table 1:** Calibration results from the simulation study with 20 centres

Method	ICC	Calibration Intercept (se)	Calibration Slope (se)
No clustering	0.05	−0.022 (0.130)	0.974 (0.077)
RE-zero	0.05	−0.008 (0.132)	0.948 (0.076)
RE-integ	0.05	−0.026 (0.129)	0.970 (0.075)
RE-approx	0.05	−0.024 (0.129)	0.968 (0.075)
GEE	0.05	−0.023 (0.131)	0.970 (0.076)
No clustering	0.10	−0.019 (0.174)	0.981 (0.073)
RE-zero	0.10	0.031 (0.182)	0.922 (0.069)
RE-integ	0.10	−0.014 (0.173)	0.984 (0.069)
RE-approx	0.10	−0.009 (0.173)	0.979 (0.068)
GEE	0.10	−0.026 (0.175)	0.983 (0.071)
No clustering	0.15	−0.016 (0.208)	0.971 (0.080)
RE-zero	0.15	0.049 (0.225)	0.884 (0.071)
RE-integ	0.15	−0.009 (0.207)	0.972 (0.073)
RE-approx	0.15	−0.004 (0.209)	0.964 (0.071)
GEE	0.15	−0.026 (0.211)	0.971 (0.076)
No clustering	0.30	0.004 (0.282)	0.963 (0.100)
RE-zero	0.30	0.142 (0.333)	0.788 (0.070)
RE-integ	0.30	−0.020 (0.280)	0.977 (0.090)
RE-approx	0.30	−0.009 (0.283)	0.956 (0.084)
GEE	0.30	−0.018 (0.285)	0.982 (0.097)

Calibration intercept and calibration slope using different forms of marginal risk calculation for varying degrees of clustering (median calibration and empirical standard errors over 100 simulated datasets)

These results replicate the findings of [3] that the incorrect marginal risk calculation, ${\widehat{\pi}}_{ij}(0)$, obtained from a random intercepts model by setting the random effects equal to zero (RE-zero) results in poorly calibrated predictions for large ICCs. In contrast, the correct marginal risk calculation, ${\widehat{\pi}}_{ij}(int)$, by integrating over the distribution of random effects (RE-integ), shows good calibration, comparable to that of standard MLE (Ignoring clustering) and GEE. Also, as speculated, the simple approximation (described by equation (4)) of marginal coefficients from the corresponding conditional ones (RE-approx) also results in good calibration in all cases. For the simulation scenario where the distribution of cluster sizes in the validation dataset differed from the distribution of cluster sizes in the training dataset the results and conclusions were similar (Additional file 1: Table S1).

**Fig. 1:**
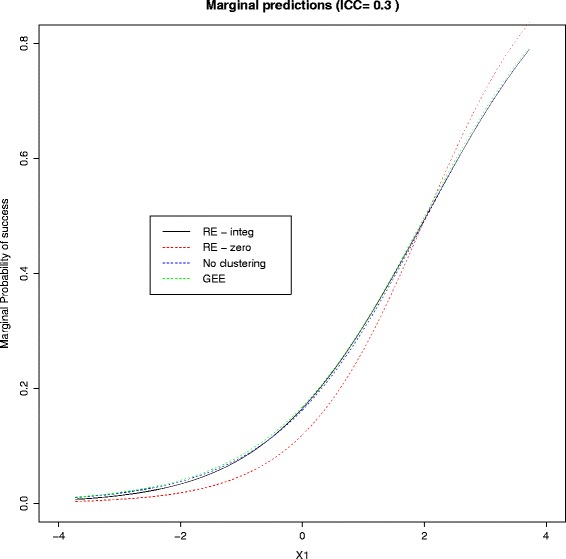
Marginal predictions for a range of values of X1, while the other predictors are set to zero

Now we demonstrate graphically the difference between the predicted risks from each method. We show the predicted risk for a range of values of the first continuous predictor, while, without loss of generality, the other five predictors are set to zero. This is done for ICC = 0.05, 0.1, 0.15 or 0.3, and the results for ICC = 0.3 are shown in Fig. 1. The naïve marginal predictions, ${\widehat{\pi}}_{ij}(0),$ tend to be more extreme than the predictions from the other methods, all of which are very similar. This is reflected by the calibration slope which is <1 for the naïve predictions but very close to one for the rest. As the ICC becomes smaller (graphs not shown) the naïve predictions approach the ones from the other methods.

## Discussion

We have compared different approaches in terms of their ability to provide correctly calibrated marginal predictions for a new individual in a new cluster. However, conditional predictions for new patients in existing clusters may also be of interest and are discussed here, along with the possibility that the random effects are not independent either of the predictors or the cluster sizes.

### Conditional predictions

Marginal predictions would be of interest for individuals who belong to clusters that were not included in the training dataset. In addition, however, predictions for new individuals who belong to clusters that were included in the training dataset may also be required. Estimates (e.g. empirical Bayes estimates) of the random effects for these clusters can be obtained during the model fitting process, and then these estimates can used to make prediction for these new individuals. This is ‘conditional’ (as opposed to marginal) prediction. In such cases, conditional predictions can be obtained by incorporating in the calculation the estimated random effects of the cluster, or, more correctly as noted by Skrondal et al. [4], by integrating over the *posterior* distribution of the random effects (see detailed expressions in the Additional file 1).

We performed additional simulations to compare the performance of these two approaches for obtaining conditional predictions in terms of calibration, and their performance was very similar (full results in the Additional file 1: Table S2 and Table S3).

### Associations between the random effects and either a predictor or the cluster size

A random intercepts model for clustered data assumes that the predictors and the cluster sizes are independent of the random effects, but either of these conditions may be violated. When a cluster-varying predictor is not independent of the random effects, ‘confounding by cluster’ [11, 12] arises. Although use of a standard random intercepts model results in a biased estimate for the effect of the predictor that is correlated with the random effect, Bouwmeester et al. [3] explained that, in fact, this correlation is beneficial in terms of prediction and particularly calibration, because inclusion of that predictor *partly* explains differences in prevalence between clusters. This was verified by their simulation studies, where the calibration of the models when a predictor, *X*, was correlated with the random intercept, appeared to be better than when there was no correlation between the predictor and the random intercept. Properly dealing with confounding, as described by Seaman et al. [11] and illustrated by Skrondal et al [4], by including the cluster-mean of *X*, as well as *X* itself, in a random intercepts model, will remove any residual confounding and produce a model suitable for prediction for patients in a new or an existing cluster. Seaman et al. [11] reviewed approaches to detect confounding by cluster. Similarly, if cluster size is not independent of the random effects, ‘informative cluster size’ [11, 13] arises. Including cluster size as a predictor will remove informative cluster size and produce a model suitable for prediction in patients in new or existing clusters.

## Conclusion

In this article we have explained why the incorrect calculation of marginalised risks from a logistic random intercepts model by setting the random effects to zero results in mis-calibrated population-average predictions. Via simulation we have shown that the correct marginalisation by integrating over the distribution of random effects solves this problem. The method of obtaining predicted risks by approximating the coefficients of the marginal model from the corresponding conditional ones also worked well. Either of the two methods could be used to compute population-average predictions, if this is desired. Although GEE would be a direct approach to obtain marginal inference, use of a random effects model would still be preferred when, for example, the covariate-dependent MCAR assumption required by GEE is likely to be violated, or when conditional predictions are required. Random effect models are consistent when data are MAR, i.e. missingness in *Y* is independent of *Y* given **X** and *u* [2] (provided that missingness does not cause any empty clusters, i.e. clusters with no observed outcomes)*.*

Arguably, in the context of clustered data where patients are clustered within hospitals, the ICC rarely exceeds 0.15. In those cases the mis-calibration of the incorrect marginalised predictions will be small. In settings where the ICC is greater than 0.15 the mis-calibration of the incorrect predictions will be a more serious issue.

In conclusion, both population-average and conditional predictions have their own use in guiding clinical decisions in different settings. In practice, a population-average prediction may be used to provide an estimate of the risk of the event for an individual that does not belong to one of the clusters used in model development. For a new individual in a cluster that was used in the model development, conditional predictions which incorporate centre-specific information by using the estimated random effect for the cluster can provide a more accurate prediction for the particular individual.

## Additional file

Additional file 1: Table S1. Calibration in the large and calibration slope for varying degrees of ICC when the distribution of the cluster sizes differs between the training and validation datasets. (mean calibration over 100 simulated datasets). **Table S2.** Overall performance measures for cluster-specific predictions for new members in existing clusters. Mean calibration intercept (standard error), median calibration slope (standard error), mean C-statistic (standard error) over 100 simulations. RE(u): Prediction is obtained by plugging in the risk formula estimates of the fixed effects and the random effect. RE(u)-int: Prediction is obtained by integrating over the posterior distribution of the random effects. **Table S3.** Overall performance measures for marginal predictions for new members in existing clusters. Mean calibration intercept (standard error), median calibration slope (standard error), mean C-statistic (standard error) over 100 simulations RE(0): Prediction is obtained by using only the fixed effects. RE-int: Prediction is obtained by integrating over the estimated distribution of the random effects.

## Abbreviations

GEE: Generalised estimation equations
ICC: Intra-cluster correlation coefficient
MLE: Maximum likelihood estimator
MCAR: Missing Completely at Random
MAR: Missing at random
