# Supplementary material for: A note on obtaining correct marginal predictions from a random intercepts model for binary outcomes
Source: BMC Med Res Methodol. 2015 Aug 5;15:59. doi: 10.1186/s12874-015-0046-6 (PMC4525751; doi:10.1186/s12874-015-0046-6)
Supplement: Additional file 1: Table S1. — Calibration in the large and calibration slope for varying degrees of ICC when the distribution of the cluster sizes differs between the training and validation datasets. (mean calibration over 100 simulated datasets). Table S2. Overall performance measures for cluster-specific predictions for new members in existing clusters. Mean calibration intercept (standard error), median calibration slope (standard error), mean C-statistic (standard error) over 100 simulations. RE(u): Prediction is obtained by plugging in the risk formula estimates of the fixed effects and the random effect. RE(u)-int: Prediction is obtained by integrating over the posterior distribution of the random effects. Table S3. Overall performance measures for marginal predictions for new members in existing clusters. Mean calibration intercept (standard error), median calibration slope (standard error), mean C-statistic (standard error) over 100 simulations RE(0): Prediction is obtained by using only the fixed effects. RE-int: Prediction is obtained by integrating over the estimated distribution of the random effects. [file 12874_2015_46_MOESM1_ESM.docx]

**Supplementary material: A note on obtaining correct marginal predictions from a random intercepts model for binary outcomes**

Menelaos Pavlou *research associate*1, Gareth Ambler *senior* *lecturer*1,

Shaun Seaman *senior statistician*2*,* Rumana Z Omar *professor*1

1 Department of Statistical Science, University College London, UK;

2 MRC Biostatistics Unit, Cambridge, UK.

**Table 1S: Calibration in the large and calibration slope for varying degrees of ICC when the distribution of the cluster sizes differs between the training and validation datasets. (mean calibration over 100 simulated datasets).**

| Method | ICC | Calibration intercept(se) | Calibration  slope (se) |
| --- | --- | --- | --- |
| No clustering | 0.05 | 0.017 (0.130) | 0.977 (0.076) |
| RE-zero | 0.05 | 0.032 (0.130) | 0.945 (0.074) |
| RE-integ | 0.05 | 0.010 (0.127) | 0.976 (0.075) |
| RE-approx | 0.05 | 0.014 (0.128) | 0.975 (0.075) |
| GEE | 0.05 | 0.012 (0.127) | 0.983 (0.076) |
| No clustering | 0.15 | 0.007 (0.176) | 0.970 (0.072) |
| RE-zero | 0.15 | 0.064 (0.192) | 0.887 (0.058) |
| RE-integ | 0.15 | 0.002 (0.177) | 0.969 (0.071) |
| RE-approx | 0.15 | 0.004 (0.178) | 0.965 (0.068) |
| GEE | 0.15 | -0.005 (0.182) | 0.971 (0.071) |
| No clustering | 0.30 | -0.020 (0.277) | 0.955 (0.093) |
| RE-zero | 0.30 | 0.121 (0.306) | 0.793 (0.066) |
| RE-integ | 0.30 | -0.003(0.261) | 0.958 (0.089) |
| RE-approx | 0.30 | 0.009 (0.263) | 0.947 (0.083) |
| GEE | 0.30 | -0.045 (0.267) | 0.984 (0.093) |

**Table 2S: Overall performance measures for cluster-specific predictions for new members in existing clusters. Mean calibration intercept (standard error), median calibration slope (standard error), mean C-statistic (standard error) over 100 simulations.** **RE(u): Prediction** **is obtained by plugging in the risk formula estimates of the fixed effects and the random effect. RE(u)-int: Prediction is obtained by integrating over the posterior distribution of the random effects.**

| Method | ICC | Calibration  intercept (se) | Calibration  slope (se) | C-statistic (se) |
| --- | --- | --- | --- | --- |
| RE(u) | 0.05 | 0.008 (0.068) | 0.978 (0.065) | 0.793 (0.004) |
| RE(u) - Int | 0.05 | 0.002 (0.067) | 0.986 (0.065) | 0.793 (0.004) |
| RE(u) | 0.10 | 0.008 (0.069) | 0.977 (0.062) | 0.802 (0.004) |
| RE(u) - Int | 0.10 | 0.001 (0.068) | 0.987 (0.062) | 0.802 (0.004) |
| RE(u) | 0.15 | 0.012 (0.070) | 0.977 (0.066) | 0.811 (0.004) |
| RE(u) - Int | 0.15 | 0.003 (0.069) | 0.988 (0.066) | 0.811 (0.004) |
| RE(u) | 0.30 | 0.016 (0.068) | 0.984 (0.065) | 0.834 (0.004) |
| RE(u) - Int | 0.30 | 0.006 (0.067) | 0.997 (0.065) | 0.834 (0.004) |

RE(u): Conditional predictions are obtained by incorporating in the calculation the estimated random intercepts of the cluster:

$$P\left( Y_{ij}=1 | \boldsymbol{X}_{ij}=\boldsymbol{x}_{ij},\hat{u}_{i} \right)=\hat{\pi}_{ij}\left( u_{i} \right)=\frac{1}{1+\exp\left( -\left[ \hat{a}_{RE}+\hat{u}_{i}+\sum_{m=1}^{p} x_{ij,m} \hat{\beta}_{RE,m} \right] \right)} , \left( 1 \right)$$

RE(u) – Int: Conditional predictions are obtained are obtained by integrating over the *posterior* distribution of the random effects:

$$\hat{\pi}_{ij}^{int}\left( u_{i} \right)=\int_{-\infty}^{\infty} \frac{1}{1+\exp\left( -\left[ \hat{a}_{RE}+u_{i}+\sum_{m=1}^{p} x_{ij,m} \hat{\beta}_{RE,m} \right] \right)}f\left( u_{i}|\boldsymbol{Y}_{1}\boldsymbol{,\ldots,}\boldsymbol{Y}_{K}\boldsymbol{,}\boldsymbol{X}_{1},\ldots,\boldsymbol{X}_{k};\hat{a}_{RE},{\hat{\boldsymbol{\beta}}}_{RE} \right) du_{i}, \left( 2 \right)$$

where $\boldsymbol{Y}_{i}$ and $\boldsymbol{X}_{i} ,i=1,\ldots,K,$ are the vector of responses and matrix of predictors, respectively, for the i*^th^* cluster.

**Table 3S: Overall performance measures for marginal predictions for new members in existing clusters. Mean calibration intercept (standard error), median calibration slope (standard error), mean C-statistic (standard error) over 100 simulations RE(0): Prediction is obtained by using only the fixed effects. RE-int: Prediction is obtained by integrating over the estimated distribution of the random effects.**

| Method | ICC | Calibration  Intercept (se) | Calibration  Slope (se) | C-statistic (se) |
| --- | --- | --- | --- | --- |
| RE(u=0) | 0.05 | 0.011 (0.070) | 0.959 (0.060) | 0.783 (0.004) |
| RE-int | 0.05 | -0.011 (0.069) | 0.987 (0.060) | 0.783 (0.004) |
| RE(u=0) | 0.10 | 0.022 (0.071) | 0.932 (0.058) | 0.780 (0.004) |
| RE-int | 0.10 | -0.023 (0.069) | 0.988 (0.060) | 0.780 (0.004) |
| RE(u=0) | 0.15 | 0.039 (0.073) | 0.907 (0.059) | 0.778 (0.004) |
| RE-int | 0.15 | -0.029 (0.069) | 0.989 (0.063) | 0.778 (0.004) |
| RE(u=0) | 0.30 | 0.092 (0.070) | 0.823 (0.053) | 0.759 (0.004) |
| RE-int | 0.30 | -0.054 (0.062) | 0.984 (0.063) | 0.759 (0.004) |

**R code for integrating over the distribution of random effects**

# Inverse logit function

#rs is the risk score (or linear predictor or predicted log-odds)

expit<-function(rs) {1/(1+exp(-rs))}

# Integrate over the distribution of u for a given patient and

#output population average predictions

papred<-function(x,beta,sigmau){

#x is an Nxp design matrix

#beta is px1 vector of estimated regression coefficients

#sigmau the standard deviation of the random effects

#Sample from the prior distribution of the random effects

u <- qnorm(seq(0.001, 0.999, 0.001))*sigmau

predu<-function(x,beta,u) {mean(expit(x%*%beta+u)) }

pa<-apply(x,1,predu,"beta"=beta,"u"=u)

return(pa)

}
